# Supplementary material for: Reduction of retinal ganglion cell death in mouse models of familial dysautonomia using AAV-mediated gene therapy and splicing modulators
Source: Sci Rep. 2023 Oct 30;13:18600. doi: 10.1038/s41598-023-45376-w (PMC10616160; doi:10.1038/s41598-023-45376-w)
Supplement: Supplementary file 1 — Supplementary Information. [file 41598_2023_45376_MOESM1_ESM.pdf]

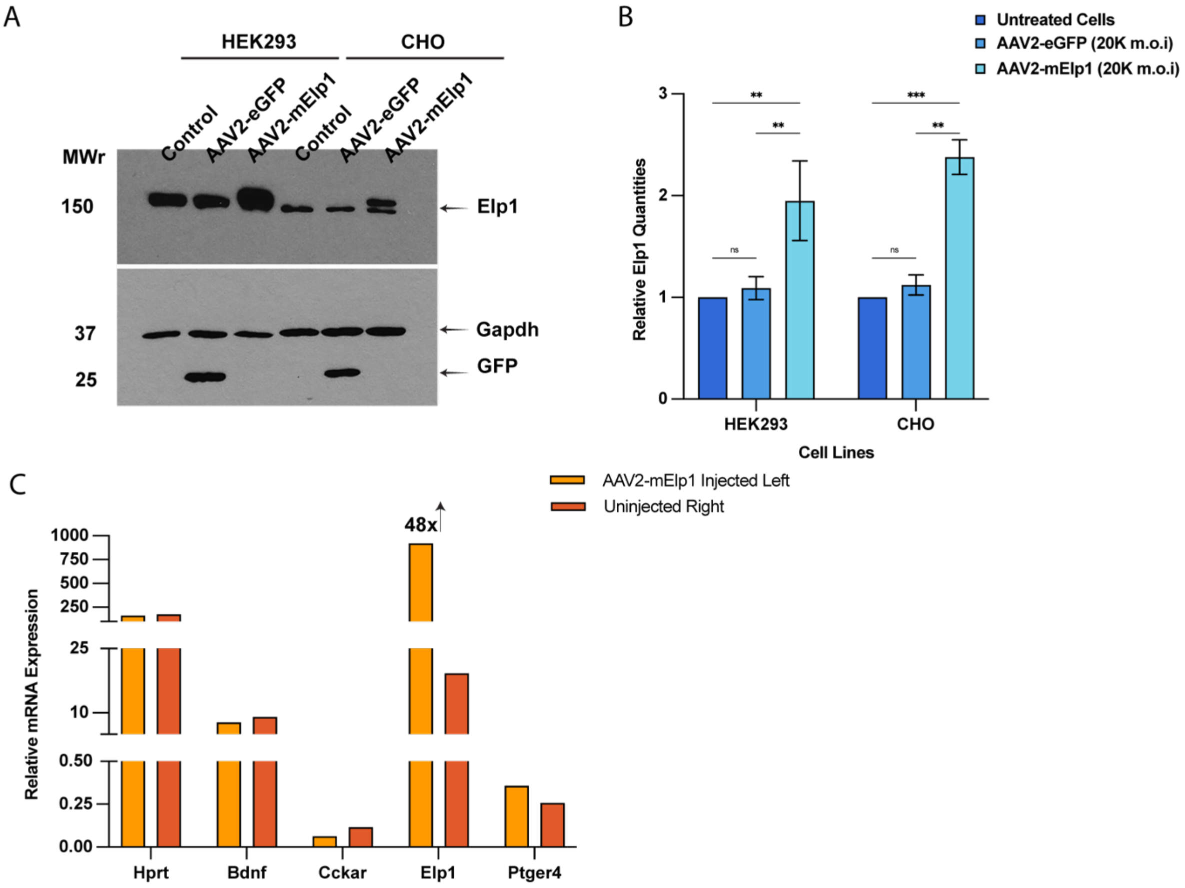

Supplemental Figure 1: (A) Representative immunoblot showing the expression of murine Elp1 in two different cell lines 4 days post-transduction with AAV2-U1a-m*Elp1* and AAV2-U1a-eGFP. This is a compiled image from two separate gels run simultaneously (see supplemental figure 3A-C for a full gel image). There is an increase in Elp1 expression in the transduced cells. 20µg of protein was loaded in each well, and GAPDH was used as the loading control. (B) Densitometric analysis of Elp1 protein in HEK293 and CHO cells transduced with AAV2-U1a-m*Elp1* compared to untreated cells (HEK293: **p = 0.004, 0.005; CHO: **p = 0.001, ***p = 0.0008, two-way ANOVA with Tukey's multiple comparisons follow-up test. The experiment was done in triplicate. (C) RT-qPCR analysis of *Elp1* expression in retinal homogenates 1 month after intravitreal injections of AAV2-U1a-m*Elp1*. Of the gene transcripts tested, only Elp1 is strongly increased.

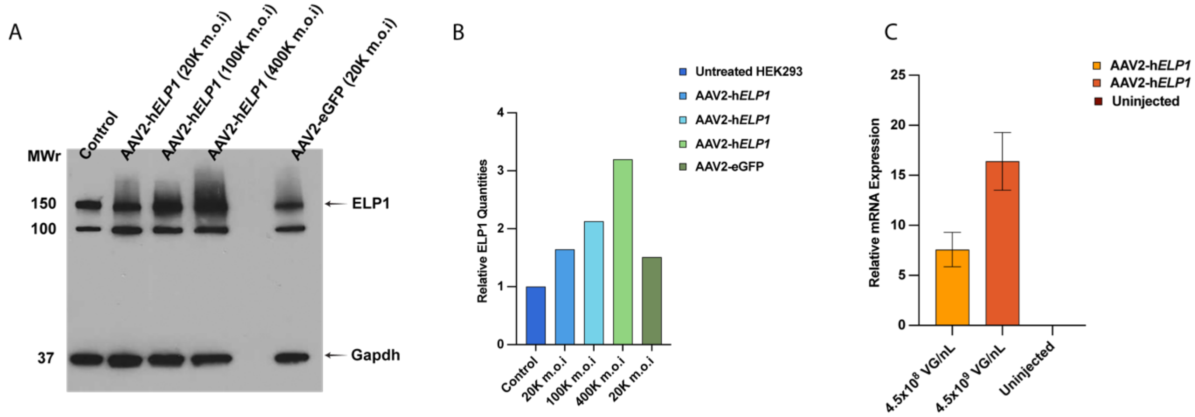

Supplemental Figure 2: (A) Representative immunoblot showing the upregulation of human ELP1 in HEK293 cells 4 days post-transduction with AAV2-U1a-h*ELP1*. Note the gradual increase in ELP1 expression as the multiplicity of infection (m.o.i.) increases. Equal amounts of protein were loaded in each well, and GAPDH was used as the loading control. This is a cropped gel image (See supplemental figure 3D for a full gel image). (B) Densitometric analysis of ELP1 protein in HEK293 cells transduced with AAV2-U1a-h*ELP1* compared to untreated cells. (C) RT-qPCR analysis of *ELP1* expression in retinal homogenates (n=2) 1 month after intravitreal injections of AAV2-U1a-h*ELP1*. As expected, we saw no expression of the human ELP1 gene in the uninjected control homogenates, whereas the control mice receiving AAV2-U1a-h*ELP1* showed increasing levels of human ELP1 protein or mRNA.

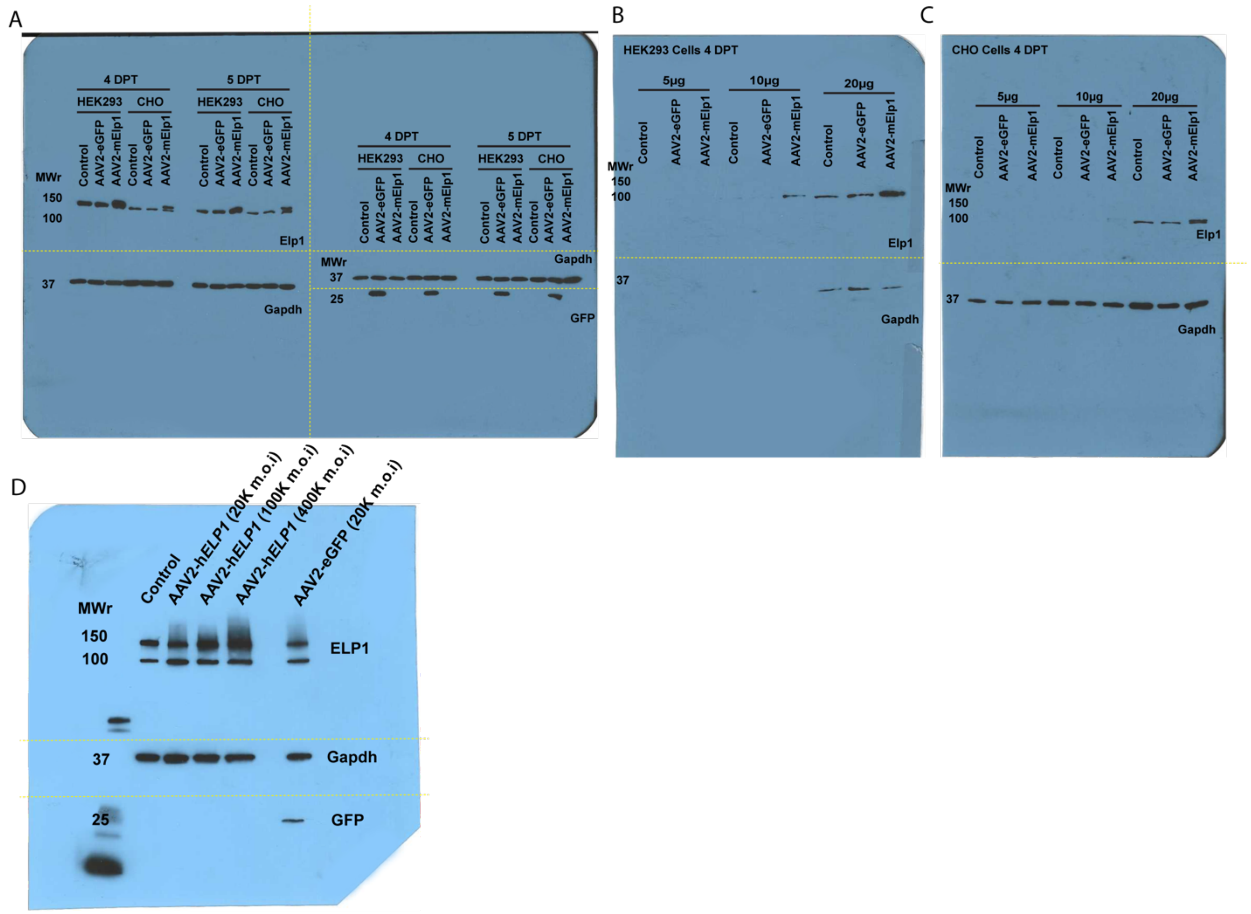

Supplemental Figure 3: Full-length western blot X-ray film for densitometric analysis of Elp1 protein expression. (A) The full gel image from supplementary figure 1 uses a 7.5% gel (left) and a 12.5% gel (right), showing the endogenous expression of murine Elp1 in HEK293 and CHO cells, which increases following infection with AAV2-mElp1 virus. *The film includes additional data that is not included in this manuscript (5 days post-transduction).* (B) A full gel image from an additional experiment using a 7.5% gel used for densitometric quantification in supplementary figure 1 shows the expression of murine Elp1 in HEK293 cells loading different protein concentrations at 4 days post-transduction. (C) A full gel image from an additional experiment using a 7.5% gel used for densitometric quantification in supplementary figure 1 shows the expression of murine Elp1 in CHO cells loading different protein concentrations at 4 days post-transduction. (D) The full gel image from supplementary figure 2 uses a 4-15% gradient gel; immunoblot shows ELP1, GAPDH, and GFP expression in HEK293 cells. For all western blot procedures, the individual membrane pieces were developed simultaneously on a single piece of X-ray film. Dotted yellow lines indicate where the PVDF membrane was cut prior to antibody hybridization.

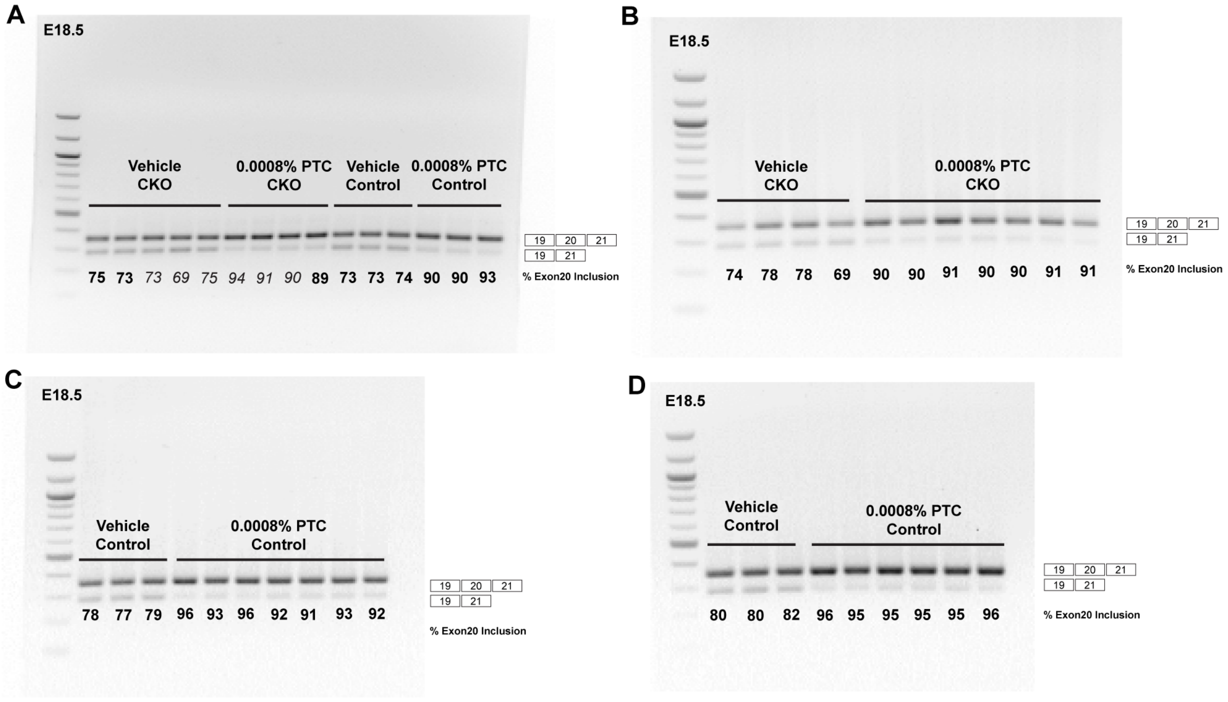

Supplemental Figure 4: ELP1 splicing analysis indicates that PTC 680 increases inclusion of exon 20. Retinae are from mice that started oral treatment of PTC680 at E18.5. All experimental samples, full-length gels, and % exon 20 inclusion are shown here. Values in red indicate samples and the portion of the gel used in Figure 4C. Samples were run on a 1.5% agarose gel stained with ethidium bromide. The gel was originally imaged on a dark background, and colors were immediately inversed for quantification and visualization. *All lanes from the original gel are included in this manuscript.*

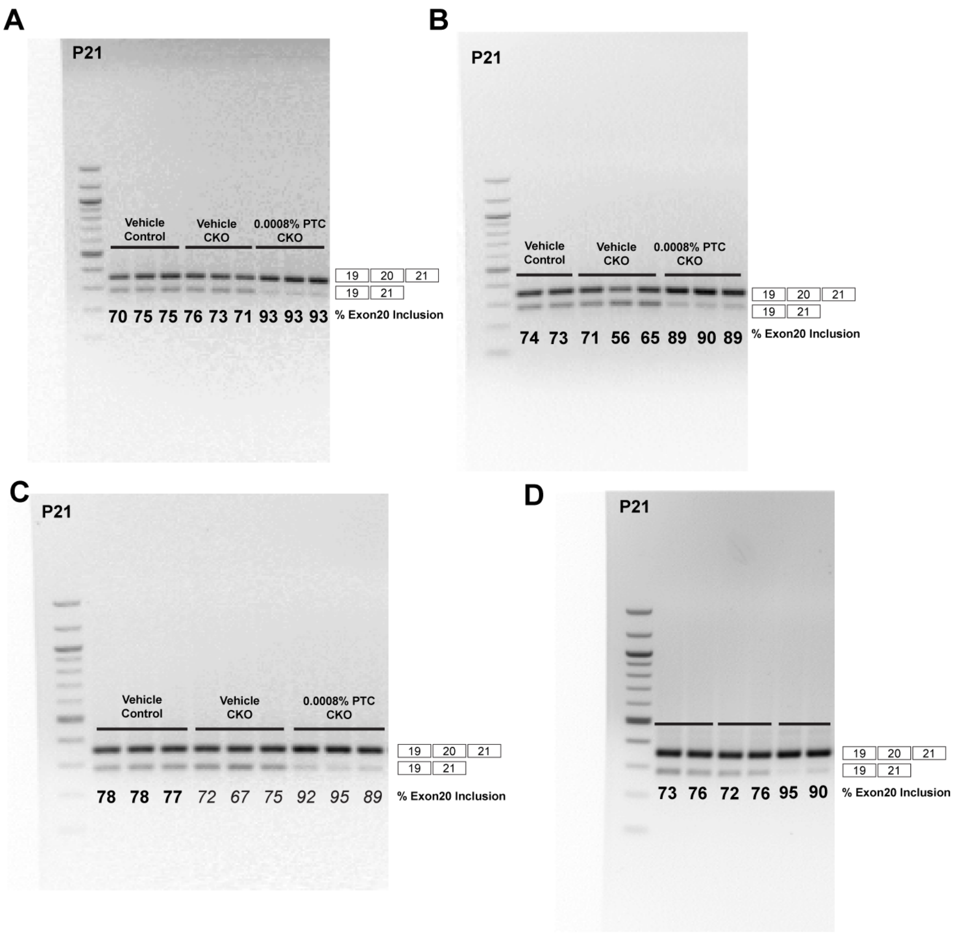

Supplemental Figure 5: ELP1 splicing analysis indicates that PTC 680 increases inclusion of exon 20. Retinae are from mice that started oral treatment of PTC680 at P21. All experimental samples, full-length gels, and % exon 20 inclusion are shown here. Values in red indicate samples and the portion of the gel used in Figure 4C. Samples were run on a 1.5% agarose gel stained with ethidium bromide. The gel was originally imaged on a dark background, and colors were immediately inversed for quantification and visualization. *5 lanes from (A,C,D) and 6 lanes from (B) were cropped off the right side because the original gel includes samples and datasets from another experiment which is not included in this manuscript.*

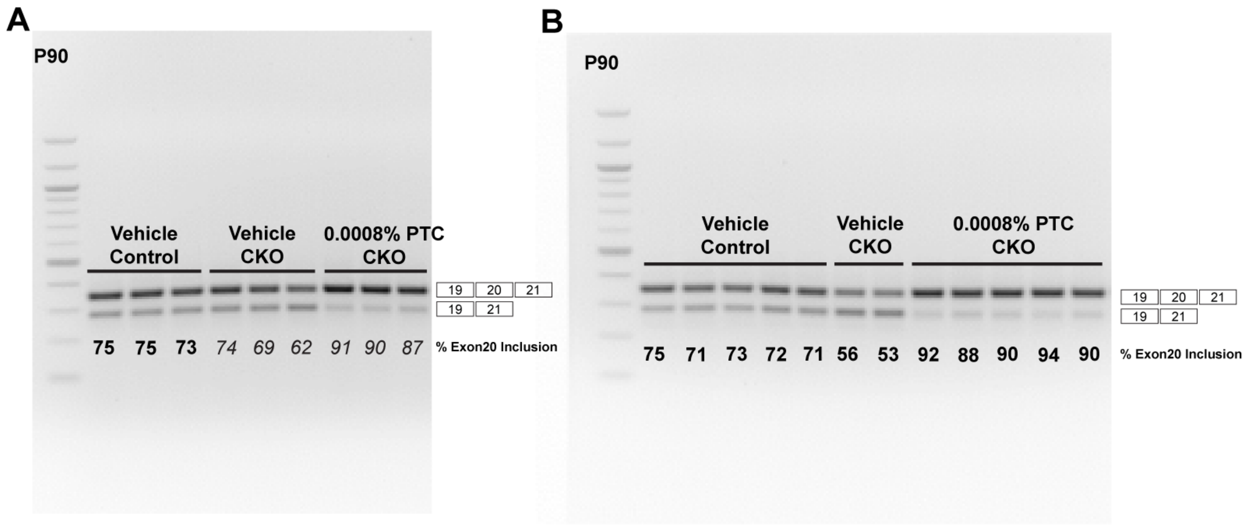

Supplemental Figure 6: ELP1 splicing analysis indicates that PTC 680 increases inclusion of exon 20. Retinae are from mice that started oral treatment of PTC680 at P90. All experimental samples, full-length gels, and % exon 20 inclusion are shown here. Values in red indicate samples and the portion of the gel used in Figure 4C. Samples were run on a 1.5% agarose gel stained with ethidium bromide. The gel was originally imaged on a dark background, and colors were immediately inversed for quantification and visualization. *6 lanes from (A) and 2 lanes from (B) were cropped off the right side because the original gel includes samples and datasets from another experiment which is not included in this manuscript.*

Supplemental Table 1: List of oligonucleotide primers

Sequence Name		Seq 5' to 3'	Accession No.
ELP1	F	CTCTGCAGTCTCAGCACACA	NM_003640.5
ELP1	R	CTGCTCCAGGATTGGCTCAA	
Elp1	F	GGTGACAGTCTTTCGGCAGA	NM_026079.3
Elp1	R	GATCAGCAGCCGGTAGGTAC	
Cckar	F	TGAACAAACGCTTTCGCCTG	NM_009827
Cckar	R	TGGCTGTAGGAATACCGGGA	
Ptger4	F	CACCACCTCGCTGAGAACTT	NM_008965
Ptger4	R	TCCTTTAGAGGCAGGCTCCT	
Hprt	F	TCAGTCAACGGGGGACATAAA	NM_013556
Hprt	R	GGGGCTGTACTGCTTAACCAG	
Actb	F	AACCCTAAGGCCAACCGTGAA	NM_007393
Actb	R	TCACGCACGATTTCCCTCTCA	
Bdnf	F	ACTGCAGTGGACATGTCTGG	NM_007540
Bdnf	R	AGTTGGCCTTTGGATACCGG	

Supplemental Table 1: Forward (F) and reverse (R) primer sequences used for RT-qPCR experiments on RNA extracted from retinal tissue.
